# Rechallenging lithium after Lithium-induced encephalopathy: A case report

**DOI:** 10.1192/j.eurpsy.2026.11217

**Published:** 2026-07-31

**Authors:** Z. Alaoui El Hassani, R. Ben Massoued, H. Khayer, P. Carrillo, J. Dhote, S. Smadja, R. Gaillard

**Affiliations:** Adult Psychiatry Department, GHU Paris Psychiatry & Neurosciences Sainte-Anne , Paris, France

## Abstract

**Introduction:**

Lithium is not only a gold-standard treatment for bipolar disorder but is also widely used in various psychiatric diseases. Lithium-induced encephalopathy is a rare but serious complication. Although this side effect is well described with high lithium blood concentrations, it is rarely described at therapeutic ranges. Moreover, rechallenging lithium after neurotoxicity remains controversial.

**Objectives:**

Describe a clinical case of lithium-induced encephalopathy occurring at therapeutic serum levels, and to evaluate the feasibility and safety of a carefully monitored rechallenging treatment with lithium.

**Methods:**

We illustrate lithium rechallenge after encephalopathy through a case of neurotoxicity occurring at therapeutic lithium ranges in a patient hospitalized in the adult psychiatric Department (SHU, sector 14), Sainte-Anne Hospital, Paris.

**Results:**

We report the case of a 53-year-old man with bipolar disorder type I, treated with lithium sustained-release (LP) 1000 mg/day and quetiapine 1200 mg/day, with lithium blood concentrations within the therapeutic range (0.76 mmol/L at day 5 and 1.05 mmol/L (at day 60). After two months of treatment, he developed rapidly progressive tremor, cognitive impairment, non-fluent (Broca’s) aphasia, lethargy, apraxia, dysarthria, and confusion. Neurological assessment revealed fine resting tremor with cogwheel rigidity, ataxic gait, and dysmetria, raising the suspicion of lithium-induced encephalopathy. Despite being in the therapeutic range (1.05 mmol/L), EEG showed diffuse theta slowing compatible with encephalopathy, and brain MRI revealed a small cortical infarct without acute lesion. Accordingly, lithium LP was discontinued, leading to clinical improvement, subsidence of neurological symptoms, and partial EEG normalization. In parallel, Quetiapine was tapered from 1200 to 300 mg/day. Therefore, a carefully monitored reintroduction of lithium immediate-release (LI), titrated up to 875 mg/day, reduced blood concentration to 0.57 mmol/L. Then, follow-up EEGs showed mild diffuse slowing without epileptiform activity. Moreover, the patient remained clinically stable, with serum lithium levels remained between 0.37–0.45 mmol/L, intra-erythrocytic lithium at 0.09 mmol/L (ratio 0.2), excluding tissue accumulation, importantly no recurrence of neurotoxic symptoms was observed.

**Conclusions:**

This case calls attention to lithium-induced encephalopathy at therapeutic index. Notably in this patient, a careful reintroduction with strict monitoring allowed stabilization without recurrence.

**Disclosure of Interest:**

None Declared

